# Daratumumab during Myeloma Induction Therapy Is Associated with Impaired Stem Cell Mobilization and Prolonged Post-Transplant Hematologic Recovery

**DOI:** 10.3390/cancers16101854

**Published:** 2024-05-13

**Authors:** Julian Mehl, Dilara Akhoundova, Ulrike Bacher, Barbara Jeker, Gaëlle Rhyner Agocs, Axel Ruefer, Susanne Soltermann, Martin Soekler, Annette Winkler, Michael Daskalakis, Thomas Pabst

**Affiliations:** 1 Department of Medical Oncology, Inselspital, Bern University Hospital, 3010 Bern, Switzerland; julian.mehl@students.unibe.ch (J.M.); dilara.akhoundovasanoyan@insel.ch (D.A.); barbara.jeker@insel.ch (B.J.); 2 Department of Hematology and Central Hematology Laboratory, Inselspital, Bern University Hospital, 3010 Bern, Switzerland; veraulrike.bacher@insel.ch (U.B.); michael.daskalakis@insel.ch (M.D.); 3 Department of Medical Oncology, HFR Fribourg-Hôpital Cantonal, 1708 Fribourg, Switzerland; gaelle.rhyner@h-fr.ch; 4 Department of Hematology, Cantonal Hospital Lucerne, 6000 Lucerne, Switzerland; axel.ruefer@luks.ch; 5 Department of Oncology and Hematology, Bürgerspital Solothurn, 4500 Solothurn, Switzerland; susanne.soltermann@spital.so.ch; 6 Department of Oncology and Hematology, Hospital Thun, 3600 Thun, Switzerland; martin.soekler@spitalstsag.ch; 7 Department of Oncology and Hematology, Biel Hospital Center, 2501 Biel, Switzerland; annette.winkler@spitalzentrum-biel.ch

**Keywords:** multiple myeloma (MM), daratumumab, anti-CD38 monoclonal antibody, mobilization, apheresis, collection, engraftment, vinorelbine, gemcitabine

## Abstract

**Simple Summary:**

Daratumumab is a CD38-targeting antibody that is being increasingly integrated into first-line multiple myeloma (MM) induction treatment, leading to an improved response depth and a longer progression-free survival. Autologous stem cell transplantation (ASCT) is commonly performed as a consolidation strategy following first-line treatment in fit MM patients. However, limited data on the short-term effects of daratumumab on the success and safety of stem cell mobilization and autologous stem cell transplantation are available to date. We analyzed the performance of stem cell mobilization and collection, as well as engraftment kinetics, in MM patients treated with daratumumab-containing induction regimens, compared to daratumumab-free therapy.

**Abstract:**

Daratumumab is being increasingly integrated into first-line multiple myeloma (MM) induction regimens, leading to improved response depth and longer progression-free survival. Autologous stem cell transplantation (ASCT) is commonly performed as a consolidation strategy following first-line induction in fit MM patients. We investigated a cohort of 155 MM patients who received ASCT after first-line induction with or without daratumumab (RVd, *n* = 110; D-RVd, *n* = 45), analyzing differences in stem cell mobilization, apheresis, and engraftment. In the D-RVd group, fewer patients successfully completed mobilization at the planned apheresis date (44% vs. 71%, *p* = 0.0029), and more patients required the use of rescue plerixafor (38% vs. 28%, *p* = 0.3052). The median count of peripheral CD34+ cells at apheresis was lower (41.37 vs. 52.19 × 10^6^/L, *p* = 0.0233), and the total number of collected CD34+ cells was inferior (8.27 vs. 10.22 × 10^6^/kg BW, *p* = 0.0139). The time to recovery of neutrophils and platelets was prolonged (12 vs. 11 days, *p* = 0.0164; and 16 vs. 14 days, *p* = 0.0002, respectively), and a higher frequency of erythrocyte transfusions (74% vs. 51%, *p* = 0.0103) and a higher number of platelet concentrates/patients were required (4 vs. 2; *p* = 0.001). The use of daratumumab during MM induction might negatively impact stem cell mobilization and engraftment in the context of ASCT.

## 1. Introduction

Autologous stem cell transplantation (ASCT) remains a key component of the first-line treatment in fit multiple myeloma (MM) patients, with recent studies showing significantly longer progression-free survival (PFS) compared to consolidation strategies without ASCT [1,2,3]. The European Haematology Association and European Society of Medical Haematology (EHA-ESMO) guidelines recommend the use of lenalidomide, bortezomib, and dexamethasone (RVd) or daratumumab, bortezomib, thalidomide, and dexamethasone (D-VTd) as first-line regimens for ASCT-eligible patients, while the Swiss guidelines recommend RVd. Both guidelines recommend melphalan-based high-dose chemotherapy (HDCT) conditioning before proceeding to ASCT and subsequent lenalidomide maintenance therapy [4,5].

However, the addition of daratumumab to newly diagnosed MM (NDMM) induction therapy is being increasingly implemented in clinical practice since recent studies have shown that daratumumab-containing regimens improve depth and duration of response compared to induction without daratumumab [6,7,8,9]. Daratumumab is a monoclonal antibody targeting CD38 and acts through a variety of direct on-tumor mechanisms as well as immune modulation [10,11,12,13,14,15]. The addition of daratumumab to RVd (D-RVd) exploits synergetic effects shown for combining daratumumab with lenalidomide and bortezomib, respectively [16,17].

Choice of induction regimen, number of administered cycles, stem cell mobilization regimen, use of plerixafor, and patient age were shown to impact CD34+ stem cell mobilization potential [18,19,20,21,22,23,24,25,26]. A higher yield of collected CD34+ cells allows for performing more than one ASCT, re-transfusion in case of engraftment failure, and administration of a higher dose of CD34+ cells, which leads to better engraftment [27,28,29]. Previous reports have shown that daratumumab might impair stem cell mobilization and collection, leading to an increased requirement for plerixafor use, impaired stem cell engraftment, and an increased complication rate during hospitalization [6,30,31,32,33,34,35,36,37,38,39,40,41]. However, current mobilization schedules, as well as standards for induction treatment, are heterogeneous among institutions. While cyclophosphamide combined with granulocyte-colony stimulating factors (G-CSF) is frequently used as mobilization therapy in many institutions, Swiss centers alternatively use vinorelbine + G-CSF or gemcitabine + G-CSF, which have both been shown to be safe and effective [20,42,43,44], with gemcitabine being preferentially used in patients with pre-existing polyneuropathy [20].

Since limited data are available on the impact of daratumumab on stem cell mobilization performance, we report data from our NDMM patient cohort treated with D-RVd versus RVd, comparing stem cell mobilization metrics as well as post-ASCT engraftment kinetics. To the best of our knowledge, this is the first study performing this comparison between patients treated with D-RVd vs. RVd, followed by vinorelbine- or gemcitabine-based mobilization therapy.

## 2. Materials and Methods

### 2.1. Patient Population and Study Endpoints

We performed a retrospective single-center case-control study. Patients were considered eligible if they were diagnosed with an NDMM as defined by the International Myeloma Working Group (IMWG) [45] and treated with first-line induction with D-RVd or RVd, followed by a consolidation with ASCT. A maximum of one cycle with a different induction regimen was permitted. Data were analyzed per intention-to-treat.

The primary endpoint was the number of circulating CD34+ cells in peripheral blood on the day of stem cell apheresis. As secondary endpoints, we assessed mobilization treatment duration, duration of apheresis, number of apheresis days, CD34+ collection yield, number of retransplanted cells, engraftment kinetics, transfusion requirements, duration of hospitalization, and infectious complications.

This study was performed following the Declaration of Helsinki and was approved by the local ethics committee of Bern, Switzerland: the Ethics Commission of the Canton of Bern (decision number #2024-00928).

### 2.2. Patient Characteristics

For basal characteristics, laboratory records between the initial diagnosis and the start of induction therapy were used. If no data were available, laboratory values up to 1 month after the start of induction were used. Laboratory values, first available later than 1 month after the start of induction therapy, were deemed “missing.” If the degree of BM infiltration was indicated with a range in reports, the mean value of the reported range was used for statistical analysis. Staging according to the revised international staging system (R-ISS) was documented whenever possible. If not available, staging according to the previously used international staging system (ISS) was reported instead [46]. High-risk cytogenetics were defined by the detection of del(17p), t(4;14), or t(14;16) via fluorescence in situ hybridization (FISH).

### 2.3. Procedures

After induction, patients underwent mobilization therapy. The following standard mobilization regimens were used: vinorelbine (35 mg/m^2^; intravenous (iv) for 10 min) or gemcitabine (1250 mg/m^2^; iv for 30 min), administered as a single infusion on day 1. Gemcitabine was used in patients with pre-existing peripheral neuropathy. Weight-adapted filgrastim (G-CSF) was added on day 4 (60 million international units (MIU) for patients ≤ 69 kg body weight, 78 MIU from 70–89 kg, and 96 MIU for patients ≥90 kg, administered subcutaneously and split into 2 doses/day) and continued until the morning of apheresis, which was routinely planned on day 8. In an alternative schedule, G-CSF was administered from day 1 until apheresis day (planned on day 5), either isolated (“G-CSF only”) or in combination with a single dose of ixazomib (4 mg, orally) on day 4. Most patients received meloxicam during mobilization therapy. Plerixafor was additionally administered on the planned apheresis day if the CD34+ peripheral concentration was <10 × 10^6^/L. Apheresis was performed when peripheral blood CD34+ cells exceeded 10–15 × 10^6^/L, aiming for a single day of collection.

While 2 × 10^6^ collected CD34+ cells/kg BW were considered a minimal requirement to proceed to ASCT, the optimal count was set at 6 × 10^6^ cells. The collected CD34+ cells were quantified after washing the apheresis product. Cell processing procedures followed local standards. Flow cytometry was used to identify CD34+ cells in the peripheral blood and quantify collected cells with a Canto-II flow cytometer (BD Biosciences, San Jose, CA, USA), following the ISHAGE guidelines [47].

HDCT regiments used before ASCT were treosulfan and melphalan (TreoMel) or isolated melphalan. TreoMel patients received treosulfan (14 mg/m^2^; iv) from day −4 to day −2 and melphalan (140 or 200 mg/m^2^, iv) on day −1 before ASCT (day 0). Patients receiving only melphalan either got a split dose (100 mg/m^2^ per day, iv) at days −2 and −1 before ASCT or 200 mg/m^2^ on day −1. In patients aged 70 and older or with a creatinine clearance between 40 and 50 mL/min, the total melphalan dose was reduced to 140 mg/m^2^. If the creatinine clearance was below 40 mL/min, the melphalan dose was adjusted to 100 mg/m^2^. The limit for transplant volume at ASCT was set at 300 mL/day with a 5% concentration of dimethyl sulfoxide (DMSO). The volume was split over several days if this limit was exceeded. Anti-infective prophylaxis consisted of sulfamethoxazole–trimethoprim, fluconazole, and valaciclovir. Dexamethasone was given to prevent engraftment syndrome. Patients were premedicated with methylprednisolone and clemastine before stem cell reinfusion. Additional comedications administered as part of supportive treatment were allopurinol, zoledronic acid, folic acid, and filgrastim (5 μg/kg/day, days +6 to +12). The time to neutrophil recovery was defined as the period between ASCT and neutrophil granulocytes exceeding 0.5 G/L. The time to platelet recovery was defined as the period between ASCT and platelets exceeding 20 G/L in the absence of platelet transfusions in the previous 3 days.

### 2.4. Therapy Response

Therapy response was reported according to the IMWG consensus criteria [48], and the limit of minimal residual disease (MRD) negativity was set at 10^−5^ (less than 1 MM cell/100,000 total cells in bone marrow aspirate).

### 2.5. Data Collection

Our institution has a register of patients receiving hematopoietic stem cell transplants (HSCT). This dataset was used to identify candidate patients. Clinical data were extracted from the “Marcell” database, which records data related to ASCT, as well as from clinical records registered in “i-pdos” and “m-pdos”.

### 2.6. Statistical Analysis

GraphPad Prism^®^ version 10 was used to create figures and perform statistical analyses, except for the multivariate analysis (MVA). *p*-values were calculated using Fisher’s exact test for categorical data, whereas the Mann–Whitney U-test was used to analyze numerical data. MVA was conducted using “R,” version 4.3.1, by performing a logistic regression model for categorical data and a linear regression model for numerical data. *p*-values below 0.05 were considered statistically significant, and percentage results were rounded to whole numbers.

## 3. Results

### 3.1. Patient Characteristics

A total of 155 consecutive patients undergoing ASCT between March 2020 and June 2023 were identified, of whom 110 received induction with RVd and 45 received D-RVd. Basal key patient characteristics are summarized in Table 1, and additional metrics are reported in Table S1. At MM diagnosis, patients in the RVd group were older, with a median age of 62 (vs. 58, *p* = 0.0317). The proportion of male patients was 65% in the RVd group and 38% in the D-RVd group (*p* = 0.0022). Paraprotein subtypes did not differ significantly (*p* = 0.3847). For 1 patient in each group, the first laboratory values were available 13 and 4 days, respectively, after the start of induction therapy. High-risk cytogenetics and initial staging did not differ significantly among both patient groups. The only parameter of documented MM diagnostic or staging criteria with a significant difference between both groups was basal LDH level (*p* = 0.0313).

### 3.2. Induction

There was no significant difference in the number of induction cycles between the RVd and the D-RVd patient groups (*p* = 0.2884). In 93% of the D-RVd patients, daratumumab was administered starting from cycles 1 (60%) or 2 (33%). One patient (1%) in the RVd group received carfilzomib and dexamethasone for 2 weeks after completion of the RVd induction. One patient (2%) in the D-RVd group received one cycle of pomalidomide, bortezomib, and dexamethasone (PVd) prior to the planned D-RVd induction. In both patients, this modification was performed due to renal function impairment. In 15% (RVd) and 16% (D-RVd) of cases, one drug from the induction combination was omitted for at least one cycle (e.g., due to intolerance). In one patient (1%) from the RVd group, two drugs were omitted for one cycle. Additional cycles of induction therapy between apheresis and HDCT were applied as “bridging” in a small percentage of both patient cohorts (4% and 13%, respectively, *p* = 0.0641). Registered parameters regarding induction therapy are summarized in Table S2.

### 3.3. Mobilization

Parameters concerning stem cell mobilization and apheresis are summarized in Table 2. Standard mobilization regimens at our institution combine a chemotherapeutic agent, most frequently vinorelbine or gemcitabine, with filgrastim. A total of 75% of patients in the RVd group and 96% in the D-RVd group received such a combination. Meloxicam was administered in the majority of cases during mobilization therapy (90% and 73% in the RVd and D-RVd groups, respectively, *p* = 0.0123). Plerixafor was used in 28% (RVd) and 38% (D-RVd) cases (*p* = 0.3052).

### 3.4. Apheresis

A total of 71% of the RVd patients could perform apheresis on the planned date vs. 44% in the D-RVd group (*p* = 0.0029). The median number of circulating peripheral CD34+ cells on the day of apheresis was lower in the D-RVd group (41.37 vs. 52.19 × 10^6^/L; *p* = 0.0233), whereas median white blood cell (WBC) counts were comparable. Nearly all patients completed the apheresis procedure in one day, with the remaining 5% (RVd) and 4% (D-RVd) completing apheresis in two days. The median apheresis time in the RVd group was 265.5 min, while it lasted 297 min for the D-RVd population (*p* = 0.0282). The total amount of CD34+ cells collected per kilogram of body weight (CD34+/kg BW) after wash was inferior in the D-RVd population (median: 8.27 vs. 10.215 × 10^6^/kg BW, *p* = 0.0139).

### 3.5. HDCT, ASCT, and Hospitalization

Key parameters regarding HDCT, ASCT, and hospitalization are summarized in Table 3; additional parameters regarding hospitalization are documented in Table S3. The majority of patients received TreoMel as HDCT: 86% in the RVd and 82% in the D-RVd cohort. Alternatively, patients received a purely melphalan-based HDCT regimen. Reinfused counts of CD34+/kg BW were 3.602 × 10^6^/kg BW (RVd) and 3.27 × 10^6^/kg BW (D-RVd), respectively (*p* = 0.0157). In a minority of patients, ASCT was administered over multiple days (5% and 9%, *p* = 0.2848). No significant differences in hospitalization duration were detected, with 23 (D-RVd) vs. 22 (RVd) days (*p* = 0.0654). The RVd group required a median of 11 days to achieve neutrophil recovery, while the D-RVd population required 12 days (*p* = 0.0164). Regarding time to platelet recovery, the RVd population achieved recovery in 14 days vs. 16 days in the D-RVd group (*p* = 0.0002). At least one platelet concentrate (PC) was administered in most patients of either group (95% vs. 98%, *p* = 0.6719). The median number of PCs administered was two (RVd) and four (D-RVd) PCs, respectively (*p* = 0.001). Erythrocyte transfusions were required during the hospitalization period in 51% (RVd) and 74% (D-RVd) of cases (*p* = 0.0103), with medians of given erythrocyte concentrates (ECs) amounting to 1 and 2 for RVd and D-RVd, respectively (*p* = 0.2837). In general, at least one infectious complication occurred in 58% (RVd) or 69% (D-RVd) of patients (*p* = 0.276). No significant difference in the incidence of any single infectious complication was documented when comparing both populations.

### 3.6. Therapy Response

Details regarding therapy responses are summarized in Table S4. The D-RVd group showed better results in all response criteria documented, with more patients achieving at least complete remission (*p* = 0.0125) and MRD negativity after ASCT (*p* = 0.0014). With a significantly shorter follow-up time for the D-RVd group, our study was not powered to generate meaningful long-term response data.

### 3.7. Multivariate Analysis

A multivariate analysis was conducted, comparing the effects of daratumumab use, age ≥65 years old, sex, (R-)ISS Staging III, and ≥VGPR after induction. The resulting *p*-values are shown in Table 4. Additional information, such as Beta or OR and 95% CI, can be found in Tables S5.1–S5.16. The significant impact of daratumumab use, age ≥65 years old, and (R-)ISS III was, in all instances, negative, impairing the respective parameters. The female sex improved results if significance was reached. ≥VGPR after induction never reached significance but was close for the parameter plerixafor use, where it also improved results.

### 3.8. Comparison of Mobilization Strategies

**Figure 1:**
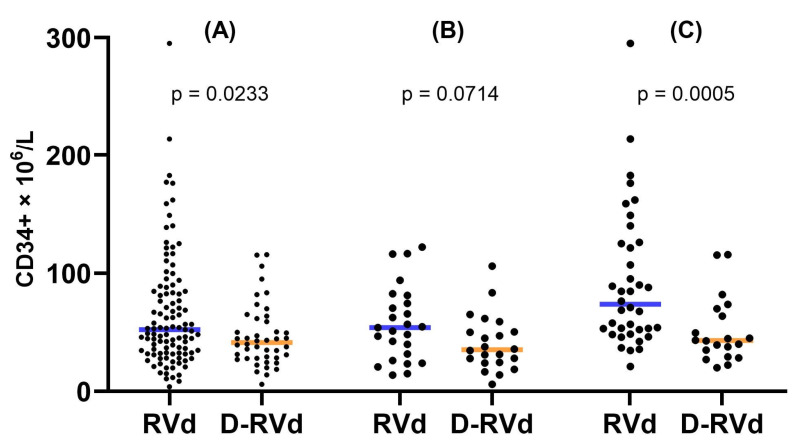
Peripheral number of CD34+ cells at the day of apheresis for the RVd and D-RVd groups; horizontal lines indicate the respective median value: (**A**) all patients; (**B**) patients mobilized with gemcitabine + G-CSF; (**C**) patients mobilized with vinorelbine + G-CSF.

Since vinorelbine- and gemcitabine-based mobilization strategies were most frequently used within both patient cohorts, we compared parameters related to stem cell mobilization, apheresis, ASCT engraftment, and post-ASCT hospitalization. Alternative mobilization regimens, used in a minority of patients, were excluded from these analyses (Tables S7 and S9). In Figure 1, results regarding our primary endpoint, the peripheral number of CD34+ cells on the day of apheresis, are displayed, comparing the impact of daratumumab addition for all patients as well as in the gemcitabine and vinorelbine subgroups. A peripheral number of CD34+ cells on the day of apheresis was consistently lower in the D-RVd cohorts, independently of the mobilization strategy. Within the D-RVd cohort, the vinorelbine subgroup performed better in the accomplishment of apheresis on the previously planned day (Table 5). Parameters regarding the comparability of the mentioned subgroups, such as age, sex, staging, etc., were documented (Tables S6, S8, and S10).

## 4. Discussion

This study retrospectively compared stem cell mobilization metrics and post-ASCT hematologic recovery of NDMM patients treated in the first line with (D-RVd) or without daratumumab (RVd). Patient characteristics within both groups were overall comparable. Slight disbalances were observed as to the patient’s age and basal LDH values. RVd-patients were a median of 4 years older at diagnosis (62 vs. 58 years, *p* = 0.0317) and had lower basal LDH values (*p* = 0.0313). There were no significant differences in MM stage distribution at diagnosis (*p* = 0.8735). A predominance of female patients has been observed among MM patients undergoing ASCT since the beginning of 2022, affecting 84% of the D-RVd patients. Both groups received a median of four induction cycles. Case-control matching was limited by a lower number of D-RVd patients due to the more recent integration of daratumumab into first-line regimens in the clinical routine.

Regarding mobilization therapy, the combination of either vinorelbine or gemcitabine with G-CSF constitutes a standard approach in Switzerland. Jeker et al. showed a 42% higher median CD34+ cell count on day +8 of mobilization when using vinorelbine instead of gemcitabine, in addition to G-CSF [20]. Our two treatment groups had significant differences in the use of mobilization therapies (Table 2, *p* < 0.0001). We addressed this by conducting a subgroup analysis between patients receiving gemcitabine + G-CSF or vinorelbine + G-CSF and comparing the performance of RVd vs. D-RVd. While lower counts of peripheral CD34+ cells were observed for the D-RVd cohort in both mobilization subgroups, vinorelbine performed better within the D-RVd cohort as to the accomplishment of apheresis on the previously planned day. Relevantly, we did not identify previous comparable studies using mobilization with gemcitabine or vinorelbine.

In our study, fewer patients in the D-RVd group were able to complete mobilization therapy on the planned apheresis date (44% vs. 71%, *p* = 0.0029). Another study reported a poorer performance of D-VTd vs. VCd as to mobilization metrics (e.g., amount of circulating peripheral CD34+ cells on apheresis day) [41]. In our study, use of plerixafor rescue was more frequent in D-RVd patients (38% vs. 28%, *p* = 0.3052), which is in line with the majority of previous reports [6,31,33,34,35,37,39,40,41]. On the contrary, Hodroj et al. reported similar rates of plerixafor usage between regimens with or without daratumumab [49]. However, most reviewed studies used induction regimens distinct from RVd/D-RVd, and stem cell mobilization procedures are overall heterogeneous across institutions.

Our primary endpoint, the number of circulating CD34+ cells on the day of apheresis, was 21% lower in the D-RVd group (41.37 vs. 52.19 × 10^6^/L, *p* = 0.0233). This was also consistently observed in the subgroup analysis: 35% lower for patients mobilized with gemcitabine (*p* = 0.0714) and 42% lower for the vinorelbine subgroup (*p* = 0.0005). Other studies reported similar results [30,31,32,37,41]. For instance, Cavallaro et al. showed lower pre-harvest concentrations of peripheral CD34+ cells in patients treated with D-VTd vs. VTd (26 vs. 76 × 10^6^/μL) [31]. Since we previously showed that meloxicam use correlates with improved stem cell mobilization [24], and both groups differed regarding this factor (*p* = 0.0123), we performed a subgroup analysis between patients mobilized with and without meloxicam. We observed no differences.

There was no difference in WBC concentrations after daratumumab exposure. In line with previous findings, WBCs were lower in the vinorelbine subgroup [20]. Almost all the patients, regardless of treatment group, completed apheresis in one day. Others’ reports showed an increase in the amount of apheresis days after treatment with daratumumab [30,33,34,36,39,40,41]. Apheresis time was longer in the D-RVd population (*p* = 0.0282), with similar results also observed in a previous report [33]. Despite significantly prolonged apheresis, 19% fewer CD34+ cells were collected (*p* = 0.0139), which is in line with numerous other studies [30,31,32,33,34,35,36,39,41]. However, other reports did not observe significant differences [37,40,49,50]. The GRIFFIN and PERSEUS trials documented lower-collected CD34+ cells in the D-RVd group [6,9]. The smaller impact of daratumumab use on collected CD34+ cells in the gemcitabine subgroup could be potentially explained by worse initial performance compared to the vinorelbine patients.

HDCT procedures were comparable. Most patients received TreoMel based on data gathered in own previous work [51]. The median amount of transplanted CD34+ cells was 9% lower for the D-RVd patients (*p* = 0.0157), which we interpreted in the context of the lower amount of collected CD34+ cells. No significant differences as to hospitalization duration (*p* = 0.0654) or splitting of ASCT were observed (9% vs. 5%, *p* = 0.2848), which was shown to complicate the hospitalization period [52]. Hospitalization duration increased by a median of 1 day in the D-RVd population (*p* = 0.0654). E. Papaiakovou et al. documented a median increase in hospitalization duration of 2 days (*p* = 0.02) [33], while Oza et al. reported no differences [36]. The time to neutrophil recovery was prolonged by a median of 1 day in the D-RVd group (12 vs. 11 days, *p* = 0.0164). Reports on the impact of daratumumab on neutrophil recovery are conflicting [6,30,31,33,36,38,39,40,50]. The time to platelet recovery was 2 days longer in the D-RVd cohort (*p* = 0.0002). Similar findings were also reported by other studies [6,31,33,36,38,39,40,50], while Zappaterra et al. documented no difference [30]. Contrarily, Mina et al. documented a median reduction of 2 days in neutrophil and platelet recovery for daratumumab patients (*p* < 0.001 and *p* = 0.001) [35]. While almost every patient received at least 1 PC, the median number of PCs administered doubled in patients treated with daratumumab induction (4 vs. 2, *p* = 0.001). Moreover, the proportion of patients receiving at least 1 EC increased significantly (51% vs. 74%, *p* = 0.0103). E. Papaiakovou et al. reported a significant increase in platelets (4 vs. 2, *p* < 0.0001) and erythrocyte units (1 vs. 0.6, *p* = 0.031) transfused in daratumumab-exposed patients [33]. Nearly all patients developed a fever during the recovery period. No differences in infectious complication rates were observed (69% vs. 58%, *p* = 0.276). Several other studies showed similar findings [31,33,36,49]. Interestingly, subgroup analysis showed differing behavior as to hospitalization duration, neutrophil recovery, number of PCs used, need for EC, and infectious complications. While gemcitabine showed worse performance when daratumumab was used in induction, the vinorelbine group was largely unaffected, possibly due to lower associated hematologic toxicity.

The definitive mechanism responsible for impaired mobilization of CD34+ cells under daratumumab exposure remains to be clarified. One mechanistic hypothesis is that CD38 is partly expressed in CD34+ stem cells, which could be contributing to daratumumab-related toxicity. However, despite the fact that the binding of daratumumab to CD34+ cells could be shown, no direct cytotoxicity on CD34+ cells has been demonstrated in vitro [53]. Moreover, CD38 expression is modifiable [54,55,56]. Hence, the use of CD38-expression modulating agents during MM treatment might also upregulate the CD38 expression on CD34+ cells, potentially leading to behavior that could not yet be replicated in vitro. Moreover, changes in adhesion of the CD34+ cells under exposure to daratumumab have been reported, which could explain the impact on mobilization performance and might even play a role in post-ASCT engraftment [57,58,59]. Further, the lower dose of retransplanted cells in daratumumab patients is likely a major factor in a more complicated engraftment period [27,28,29]. The clonogenic potential of hematopoietic stem cells might be influenced by daratumumab, with a reported negative impact on the burst-forming unit-erythroid (BFU-E) [30]. This effect could not be observed by another group, although with notably different underlying conditions [50].

The multivariate analysis showed a negative impact of age over 65 on several assessed parameters, including the primary endpoint. As previously highlighted, the D-RVd group was younger in median (58 vs. 62 y.o., *p* = 0.0317) and fewer patients were ≥65 years old (27% vs. 37%, *p* = 0.2636). Therefore, we believe that age had no relevant impact on impaired stem cell mobilization and engraftment in the D-RVd population. Female sex was associated with a shorter time to platelet recovery (*p* = 0.042) and a lower number of PC transfused (*p* = 0.055). Since a higher proportion of D-RVd patients were female (62% vs. 35%, *p* = 0.0022), patient sex distribution could have acted as a potential confounder.

We compared mobilization and engraftment parameters in D-RVd patients mobilized either with gemcitabine or vinorelbine. Overall, vinorelbine outperformed gemcitabine in the analyzed parameters, lining up with previous reports concerning daratumumab-free regimens [20]. A higher percentage of patients treated with D-RVd and mobilized with vinorelbine + GCSF could complete mobilization on the planned date, compared to gemcitabine + G-CSF (62% vs. 27%, *p* = 0.0329), had shorter times to neutrophil and platelet recovery, and required fewer transfusions. These results suggest that mobilization with vinorelbine might positively impact stem cell mobilization and post-ASCT hematologic recovery, as compared to gemcitabine, in patients treated with daratumumab.

Therapy response was improved with daratumumab addition to RVd, in line with the GRIFFIN and PERSEUS trials, underscoring the relevance of daratumumab-based combinations in the treatment of MM [6,9]. More patients achieved a CR (*p* = 0.0125) and MRD negativity after ASCT (*p* = 0.0014).

The retrospective design of the study and the disbalance in sample size between the D-RVd and RVd groups limit optimal comparability between both patient cohorts. Further research on the impact of daratumumab on ASCT short- and long-term outcomes, as well as optimization of mobilization strategies in patients treated with daratumumab, would be needed.

## 5. Conclusions

The addition of daratumumab to first-line induction with RVd was associated with 21% fewer circulating CD34+ cells detected on the day of apheresis (*p* = 0.0233), lower counts of collected and reinfused CD34+ cells (*p* = 0.0139 and *p* = 0.0157), and more frequent use of plerixafor rescue. Apheresis was delayed more frequently within the D-RVd patient cohort (*p* = 0.0029). Moreover, longer times to neutrophil and platelet recovery (*p* = 0.0164 and *p* = 0.0002), a higher number of transfused platelet concentrates (*p* = 0.001), and an increased need for erythrocyte concentrates (*p* = 0.0103) were documented in the D-RVd cohort. Further research would be required to optimize stem cell mobilization in MM patients receiving daratumumab-containing regimens.

## Figures and Tables

**Table 1:** Patient basal characteristics at diagnosis of MM.

Parameter	RVd (*n* = 110)	D-RVd (*n* = 45)	*p*-Value
Age at diagnosis (y), median (range)	62 (31–75)	58 (41–75)	0.0317
Male sex, *n* (%)	72 (65)	17 (38)	0.0022
FISH, *n* (%) ^a^			0.5296
High-risk cytogenetics	23 (28)	14 (35)	
Non-high risk	59 (72)	26 (65)	
(R-)ISS, *n* (%) ^b^			0.8744
I	30 (28)	14 (31)	
II	50 (47)	19 (42)	
III	27 (25)	12 (27)	

Data missing for *n* patients (RVd/D-RVd): ^a^ (28/5), ^b^ (3/0).

**Table 2:** Mobilization and apheresis.

Parameter	RVd (*n* = 110)	D-RVd (*n* = 45)	*p*-Value
Mobilization medication, *n* (%)			<0.0001
Vinorelbine + G-CSF	41 (37)	21 (47)	
Gemcitabine + G-CSF	27 (25)	22 (49)	
Ixazomib + G-CSF	15 (14)	0 (0)	
G-CSF only	27 (25)	2 (4)	
Mobilization without meloxicam, *n* (%)	11 (10)	12 (27)	0.0123
Plerixafor used, *n* (%) ^a^	27 (28)	15 (38)	0.3052
Apheresis on the planned date, *n* (%) ^b^	77 (71)	20 (44)	0.0029
Mobilization days until apheresis (d), median (range) ^b^	8 (8–10)	9 (8–10)	0.0006
Measurements on day of apheresis:			
CD34+ × 10^6^/L, median (range) ^c^	52.19 (3.85–295.14)	41.37 (6.05–115.6)	0.0233
WBC × 10^9^/L, median (range) ^d^	34.33 (8.97–80.34)	33.07 (16.9–75.49)	0.9526
CD34+/WBC (%), median (range) ^d^	0.16 (0.02–0.83)	0.13 (0.02–0.45)	0.0463
Apheresis time (min), median (range) ^e^	265.5 (99–724)	297 (158–1000)	0.0282
Apheresis in one day, *n* (%)	104 (95)	43 (96)	>0.9999
CD34+ × 10^6^/kg BW, median (range)	10.22 (2.39–41.54)	8.27 (3.26–17.37)	0.0139

Data missing for *n* patients (RVd/D-RVd): ^a^ (14/6), ^b^ (2/0), ^c^ (5/1), ^d^ (9/4), ^e^ (8/6).

**Table 3:** HDCT, ASCT, and Hospitalization.

Parameter	RVd (*n* = 110)	D-RVd (*n* = 45)	*p*-Value
HDCT, *n* (%)			0.619
Treosulfan/Melphalan	95 (86)	37 (82)	
Melphalan	15 (14)	8 (18)	
Transp. CD34+ × 10^6^/kg BW, median (range)	3.6 (2.05–10.36)	3.27 (1.90–5.15)	0.0157
ASCT on multiple days, *n* (%)	5 (5)	4 (9)	0.2848
Hospitalization duration (d), median (range)	22 (13–51)	23 (18–39)	0.0654
Time to neutrophil recovery (d), median (range) ^a^	11 (9–27)	12 (10–20)	0.0164
Time to platelet recovery (d), median (range) ^b^	14 (11–20)	16 (11–27)	0.0002
≥1 PC used, *n* (%) ^c^	104 (95)	44 (98)	0.6719
Number of PCs used, median (range) ^d^	2 (1–16)	4 (1–19)	0.001
≥1 EC used, *n* (%) ^e^	54 (51)	32 (74)	0.0103
Number of ECs used, median (range) ^f^	1 (1–19)	2 (1–8)	0.2837
Fever during hospitalization, *n* (%)	106 (96)	44 (98)	>0.9999
≥1 Infectious complication, *n* (%)	64 (58)	31 (69)	0.276

Data missing for *n* patients (RVd/D-RVd): ^a^ (1/0), ^b^ (9/1), ^c^ (1/0), ^d^ (4/0), ^e^ (4/2), ^f^ (12/6).

**Table 4:** Impact of treatment with daratumumab, age, sex, (R-)ISS stage, and response to induction treatment on stem cell mobilization, collection, and engraftment, multivariate analysis.

**Parameter**	**Dara Used**	**Age ≥65**	**Sex (f)**	**(R-)ISS III**	**≥** **VGPR**
Apheresis on planned date	**0.001**	0.11	0.4	0.5	0.8
Mobilization duration	**<0.001**	0.074	0.6	0.6	0.8
Plerixafor use	0.2	**0.021**	0.3	>0.9	0.054
CD34+ × 10^6^/L	**0.004**	**0.001**	0.6	0.2	0.4
WBC	0.8	0.7	**0.018**	0.4	0.5
Apheresis time	**0.012**	0.3	**0.034**	0.8	0.5
Collected CD34+	**0.007**	**<0.001**	0.8	0.067	0.8
Transplanted CD34+	**0.010**	>0.9	0.4	**0.012**	0.5
Hospitalization	0.2	**0.048**	0.6	0.7	0.2
Neutrophil recovery	0.069	0.2	0.13	0.4	0.11
Platelet recovery	**<0.001**	0.5	**0.042**	0.8	0.8
≥1 PC used	0.3	0.3	0.7	0.7	>0.9
Nr. of PCs used	**<0.001**	0.2	0.055	>0.9	0.5
≥1 EC used	0.058	0.5	0.3	0.2	0.6
Nr. of ECs used	0.2	0.14	0.4	0.7	0.4
≥1 infectious complication	0.12	0.088	0.7	0.3	0.8

Significant *p*-values are highlighted in bold.

**Table 5:** D-RVd/gemcitabine + G-CSF vs. D-RVd/vinorelbine + G-CSF.

Parameter	Gemcitabine (*n* = 22)	Vinorelbine (*n* = 21)	*p*-Value
Apheresis on the planned date, *n* (%)	6 (27)	13 (62)	0.0329
Mobilization days until apheresis (d), median (range)	9 (8–10)	8 (8–9)	0.0070
Plerixafor used, *n* (%) ^a^	10 (50)	4 (24)	0.1734
Measurements on day of apheresis:			
CD34+ × 10^6^/L, median (range) ^b^	35.23 (6.05–106)	43.03 (19.97–115.6)	0.2174
WBC × 10^9^/L, median (range) ^c^	37.03 (18.41–75.49)	26.82 (16.9–52.32)	0.0067
CD34+/WBC (%), median (range) ^c^	0.1 (0.02–0.27)	0.16 (0.08–0.45)	0.0071
Apheresis time (min), median (range) ^d^	359.5 (192–1000)	277 (158–460)	0.0200
Coll. CD34+ × 10^6^/kg BW, median (range)	8.16 (3.26–13.66)	8.27 (3.6–17.37)	0.7093
Transp. CD34+ × 10^6^/kg BW, median (range)	3.16 (1.9–4.98)	3.3 (2–5.15)	0.8569
Hospitalization duration (d), median (range)	24.5 (19–39)	22 (18–35)	0.2470
Time to neutrophil recovery (d), median (range)	12 (10–20)	11(10–12)	0.0158
Time to platelet recovery (d), median (range) ^e^	16 (13–27)	15 (11–25)	0.0430
≥1 PC used, *n* (%)	22 (100)	20 (95)	0.4884
Number of PCs used, median (range)	5 (1–19)	2.5 (1–8)	0.0015
≥1 EC used, *n* (%) ^f^	19 (90)	11 (55)	0.0148
Number of ECs used, median (range) ^g^	2 (1–8)	1 (1–4)	0.3921
Fever during hospitalization, *n* (%)	22 (100)	20 (95)	0.4884
≥1 Infectious complication, *n* (%)	16 (73)	13 (62)	0.5256

Data missing for *n* patients (gemcitabine/vinorelbine): ^a^ (2/4), ^b^ (0/1), ^c^ (1/3), ^d^ (4/2), ^e^ (1/0), ^f^ (1/1), ^g^ (3/3).

## Data Availability

Data are available on request due to restrictions, privacy, and ethics.

## Supplementary Materials

The following supporting information can be downloaded at https://www.mdpi.com/article/10.3390/cancers16101854/s1, Table S1: Additional patient characteristics; Table S2: Induction therapy; Table S3: Infectious complications, germs documented, and death during hospitalization; Table S4: Response to therapy; Table S5.1: Duration of mobilization therapy, linear regression model; Table S5.2: Mobilization completed on the planned date, logistic regression model; Table S5.3: Plerixafor use, logistic regression model; Table S5.4: Number of peripheral CD34+ cells at day of apheresis, linear regression model; Table S5.5: White blood cells at day of apheresis, linear regression model; Table S5.6: Apheresis time, linear regression model; Table S5.7: Collected CD34+ cells, linear regression model; Table S5.8: Transplanted CD34+ cells, linear regression model; Table S5.9: Hospitalization duration, linear regression model; Table S5.10: Time to neutrophil recovery, linear regression model; Table S5.11: Time to platelet recovery, linear regression model; Table S5.12: ≥1 platelet concentrate used (yes/no), logistic regression model; Table S5.13: Number of platelet concentrates given, linear regression model; Table S5.14: ≥1 erythrocyte concentrate used (yes/no), logistic regression model; Table S5.15: Number of erythrocyte concentrates given, linear regression model; Table S5.16: ≥1 infectious complication (yes/no), logistic regression model; Table S6: Basal data RVd vs. D-RVd with gemcitabine + G-CSF; Table S7: Parameters RVd vs. D-RVd with gemcitabine + G-CSF; Table S8: Basal data RVd vs. D-RVd with vinorelbine + G-CSF; Table S9: RVd vs. D-RVd with vinorelbine + G-CSF; Table S10: Basal data D-RVd patients with gemcitabine + G-CSF vs. vinorelbine + G-CSF.
