# Supplementary material for: Daratumumab during Myeloma Induction Therapy Is Associated with Impaired Stem Cell Mobilization and Prolonged Post-Transplant Hematologic Recovery
Source: Cancers (Basel). 2024 May 13;16(10):1854. doi: 10.3390/cancers16101854 (PMC11119719; doi:10.3390/cancers16101854)
Supplement: Supplementary file 1 [file cancers-16-01854-s001.zip › cancers-3009628-supplementary.pdf]

cancers MDPI

## Supplementary Material

**Table S1.** Additional basal patient characteristics.

**Copyright:** © 2024 by the authors. Licensee MDPI, Basel, Switzerland. This article is an open access article distributed under the terms and conditions of the Creative Commons Attribution (CC BY) license (https://creativecommons.org/licenses/by/4.0/).

| Parameter | RVd (*n*=110) | D-RVd (*n*=45) | *p*-Value |
|---|---|---|---|
| Paraprotein | | | 0.3847 |
| IgG, *n* (%) | 74 (67) | 26 (58) | |
| IgA, *n* (%) | 15 (14) | 11 (24) | |
| IgM, *n* (%) | 1 (1) | 0 (0) | |
| Light chain only, *n* (%) | 20 (18) | 8 (18) | |
| Light-chain type, *n* (%) | | | >0.9999 |
| Lambda | 36 (33) | 15 (33) | |
| Kappa | 74 (67) | 30 (67) | |
| MM diagnostic criteria | | | |
| Hypercalcaemia (>2,6 mmol/L), *n* (%)[a] | 16 (17) | 9 (21) | 0.6337 |
| Renal insufficiency (GFR <89ml/min), *n* (%)[b] | 26 (27) | 15 (36) | 0.4185 |
| Creatinin (µmol/L), median (range)[c] | 82 (44-770) | 82 (46-1085) | 0.6225 |
| Anaemia (<110 g/L), *n* (%)[d] | 40 (43) | 26 (59) | 0.1011 |
| Hb (g/L), median (range)[e] | 112 (70-158) | 104.5 (72-151) | 0.0665 |
| BM infiltration detected, *n* (%)[f] | 107 (99) | 43 (98) | 0.4965 |
| Degree of BM infiltration, median (range)[g] | 60 (2.3-100) | 50 (5-100) | 0.9537 |
| Osteolytic lesions, *n* (%)[h] | 83 (78) | 34 (77) | >0.9999 |
| MM staging criteria | | | |
| β2-microglobulin >3.5 mg/L, *n* (%)[i] | 33 (36) | 23 (53) | 0.0641 |
| Albumin <35 g/L, *n* (%)[j] | 35 (44) | 16 (42) | >0.9999 |
| LDH U/L, median (range)[k] | 214.5 (60-630) | 185 (115-487) | 0.0313 |

Data missing for *n* patients (RVd/D-RVd): [a](17/3), [b](15/3), [c](21/8), [d](18/1), [e](20/3), [f](2/1), [g](4/1), [h](4/1), [i](19/2), [j](30/7), [k](34/8).

**Table S2.** Induction therapy.

| Parameter | RVd (*n*=110) | D-RVd (*n*=45) | *p*-Value |
|---|---|---|---|
| Cycles before apheresis, *n* (%) | | | 0.2884 |
| 3 | 28 (25) | 6 (13) | |
| 4 | 77 (70) | 38 (84) | |
| 5 | 4 (4) | 1 (2) | |
| 6 | 1 (1) | 0 (0) | |
| Start of Daratumumab (Cycle), *n* (%) | | | |
| 1 | - | 27 (60) | |
| 2 | - | 15 (33) | |
| 3 | - | 2 (4) | |
| 4 | - | 1 (2) | |
| Alt. induction for (less than) 1 cycle, *n* (%) | 1 (1) | 1 (2) | 0.4977 |
| Pat. with ≥1 C minus 1 Drug, *n* (%) | 16 (15) | 7 (16) | >0.9999 |
| Number of cycles, median (range) | 1 (1-3) | 1 (1-2) | 0.8218 |
| Pat. with ≥1C minus 2 Drugs, *n* (%) | 1 (1) | 0 (0) | >0.9999 |
| Number of cycles, median (range) | 1 (1-1) | 0 (0) | >0.9999 |
| Bridging cycles between apheresis and HDCT, *n* (%) | 4 (4) | 6 (13) | 0.0641 |
| Number of cycles, median (range) | 2 (1-3) | 2 (1-4) | 0.7524 |

Abbreviations: Alt.=Alternative; Pat.=Patients; C=Cycle

**Table S3.** Infectious complications, microbiological agents documented and death during hospitalization.

| Parameter | RVd (*n*=110) | D-RVd (*n*=45) | *p*-Value |
|---|---|---|---|
| Infectious complications, *n* (%) | | | |
| ≥1 infectious complication | 64 (58) | 31 (69) | 0.276 |
| Bacteremia | 32 (29) | 16 (36) | 0.4487 |
| Catheter-associated infect | 18 (16) | 7 (16) | >0.9999 |
| Pneumonia | 7 (6) | 4 (9) | 0.7311 |
| Sepsis | 3 (3) | 2 (4) | 0.6281 |
| Septic shock | 1 (1) | 1 (2) | 0.4977 |
| Septic thrombosis | 2 (2) | 0 (0) | >0.9999 |
| Urinary tract infection | 6 (5) | 3 (7) | 0.7195 |
| C. difficile colitis | 3 (3) | 0 (0) | 0.5568 |
| Panaritium cutaneum | 1 (1) | 1 (2) | 0.4977 |
| Oral candidiasis | 5 (5) | 3 (7) | 0.6917 |
| Candida esophagitis | 1 (1) | 0 (0) | >0.9999 |
| Stomatitis aphtosa | 1 (1) | 0 (0) | >0.9999 |
| Abscess | 2 (2) | 0 (0) | >0.9999 |
| Erysipelas | 1 (1) | 0 (0) | >0.9999 |
| Germ detection in urine culture | 31 (28) | 18 (40) | 0.1834 |
| Germs, *n* (%) | | | |
| Detection of ≥1 germ | 57 (52) | 29 (64) | 0.1599 |
| Escherichia coli | 15 (14) | 8 (18) | 0.619 |
| Clostridioides difficile | 3 (3) | 0 (0) | 0.5568 |
| Coagulase-negative staphylococci | 28 (25) | 8 (18) | 0.4027 |
| Streptococcus viridans | 7 (6) | 2 (4) | >0.9999 |
| Enterococcus faecium | 3 (3) | 3 (7) | 0.3573 |
| Klebsiella pneumoniae | 3 (3) | 0 (0) | 0.5568 |
| Pseudomonas aeruginosa | 0 (0) | 3 (7) | 0.0233 |
| Viruses | 14 (13) | 10 (22) | 0.1486 |
| Fungi | 1 (1) | 3 (7) | 0.0739 |
| Other germs | 10 (9) | 3 (7) | 0.7575 |
| Death, n (%) | 1 (1) | 1 (2) | 0.4977 |

**Table S4.** Response to therapy.

| Parameter | RVd (n=110) | D-RVd (n=45) | *p*-Value |
|---|---|---|---|
| Response after induction, *n* (%)[a] | | | 0.1113 |
| CR | 22 (22) | 9 (22) | |
| VGPR | 43 (43) | 24 (59) | |
| PR | 35 (35) | 8 (20) | |
| SD | 1 (1) | 0 (0) | |
| PD | 0 (0) | 0 (0) | |
| ≥VGPR after induction, *n* (%)[a] | 65 (64) | 33 (80) | 0.0724 |
| Response after ASCT, *n* (%)[b] | | | 0.0029 |
| sCR | 52 (48) | 35 (80) | |
| CR | 30 (28) | 6 (14) | |
| VGPR | 14 (13) | 3 (7) | |
| PR | 12 (11) | 0 (0) | |
| SD | 0 (0) | 0 (0) | |
| PD | 1 (1) | 0 (0) | |
| ≥CR after ASCT, *n* (%)[b] | 82 (75) | 41 (93) | 0.0125 |
| MRD negativity, *n* (%)[c] | 64 (62) | 37 (88) | 0.0014 |

Data missing for *n* patients (RVd/D-RVd): [a](9/4), [b](1/1), [c](6/3).

**Table S5.1.** Duration of mobilization therapy, linear regression model.

| Parameter | Beta | 95% CI | *p*-Value |
|---|---|---|---|
| Daratumumab used | 0.38 | 0.16, 0.60 | <0.001 |
| Age ≥65 years | 0.19 | -0.02, 0.39 | 0.074 |
| Female sex | -0.05 | -0.26, 0.15 | 0.6 |
| (R-)ISS Stage III | -0.06 | -0.28, 0.17 | 0.6 |
| ≥VGPR after induction | -0.03 | -0.25, 0.19 | 0.8 |

**Table S5.2.** Mobilization completed on the planned date, logistic regression model.

| Parameter | OR | 95% CI | *p*-Value |
|---|---|---|---|
| Daratumumab used | 0.26 | 0.11, 0.58 | 0.001 |
| Age ≥65 years | 0.53 | 0.24, 1.14 | 0.11 |
| Female sex | 1.43 | 0.65, 3.24 | 0.4 |
| (R-)ISS Stage III | 1.37 | 0.58, 3.34 | 0.5 |
| ≥VGPR after induction | 0.91 | 0.39, 2.08 | 0.8 |

**Table S5.3.** Plerixafor use, logistic regression model.

| Parameter | OR | 95% CI | *p*-Value |
|---|---|---|---|
| Daratumumab used | 1.84 | 0.72, 4.72 | 0.2 |
| Age ≥65 years | 2.73 | 1.17, 6.51 | 0.021 |
| Female sex | 1.53 | 0.64, 3.70 | 0.3 |
| (R-)ISS Stage III | 0.99 | 0.37, 2.55 | >0.9 |
| ≥VGPR after induction | 0.41 | 0.17, 1.01 | 0.054 |

**Table S5.4.** Number of peripheral CD34+ cells at day of apheresis, linear regression model.

| Parameter | Beta | 95% CI | *p*-Value |
|---|---|---|---|
| Daratumumab used | -25 | -42, -8.0 | 0.004 |
| Age ≥65 years | -26 | -42, -11 | 0.001 |
| Female sex | 4.3 | -12, 20 | 0.6 |
| (R-)ISS Stage III | -10 | -27, 7.0 | 0.2 |
| ≥VGPR after induction | 6.5 | -10, 23 | 0.4 |

**Table S5.5.** White blood cells at day of apheresis, linear regression model.

| Parameter | Beta | 95% CI | *p*-Value |
|---|---|---|---|
| Daratumumab used | -0.73 | -6.0, 4.5 | 0.8 |
| Age ≥65 years | -0.82 | -5.6, 4.0 | 0.7 |
| Female sex | 6.0 | 1.0, 11 | 0.018 |
| (R-)ISS Stage III | 2.2 | -3.1, 7.4 | 0.4 |
| ≥VGPR after induction | -1.7 | -6.8, 3.4 | 0.5 |

**Table S5.6.** Apheresis time, linear regression model.

| Parameter | Beta | 95% CI | *p*-Value |
|---|---|---|---|
| Daratumumab used | 63 | 14, 112 | 0.012 |
| Age ≥65 years | 23 | -22, 68 | 0.3 |
| Female sex | -50 | -97, -3.7 | 0.034 |
| (R-)ISS Stage III | 5.9 | -46, 57 | 0.8 |
| ≥VGPR after induction | -15 | -63, 33 | 0.5 |

**Table S5.7.** Collected CD34+ cells, linear regression model.

| Parameter | Beta | 95% CI | *p*-Value |
|---|---|---|---|
| Daratumumab used | -2.4 | -4.2, -0.68 | 0.007 |
| Age ≥65 years | -3.1 | -4.7, -1.5 | <0.001 |
| Female sex | 0.21 | -1.4, 1.9 | 0.8 |
| (R-)ISS Stage III | -1.7 | -3.5, 0.12 | 0.067 |
| ≥VGPR after induction | -0.23 | -2.0, 1.5 | 0.8 |

**Table S5.8.** Transplanted CD34+ cells, linear regression model.

| Parameter | Beta | 95% CI | *p*-Value |
|---|---|---|---|
| Daratumumab used | -0.60 | -1.1, -0.15 | 0.010 |
| Age ≥65 years | -0.02 | -0.44, 0.40 | >0.9 |
| Female sex | 0.19 | -0.23, 0.62 | 0.4 |
| (R-)ISS Stage III | -0.58 | -1.0, -0.13 | 0.012 |
| ≥VGPR after induction | 0.15 | -0.29, 0.59 | 0.5 |

**Table S5.9.** Hospitalization duration, linear regression model.

| Parameter | Beta | 95% CI | *p*-Value |
|---|---|---|---|
| Daratumumab used | 1.5 | -0.56, 3.6 | 0.2 |
| Age ≥65 years | 1.9 | 0.02, 3.8 | 0.048 |
| Female sex | 0.56 | -1.4, 2.5 | 0.6 |
| (R-)ISS Stage III | -0.38 | -2.5, 1.7 | 0.7 |
| ≥VGPR after induction | -1.3 | -3.3, 0.74 | 0.2 |

**Table S5.10.** Time to neutrophil recovery, linear regression model.

| Parameter | Beta | 95% CI | *p*-Value |
|---|---|---|---|
| Daratumumab used | 0.63 | -0.05, 1.3 | 0.069 |
| Age ≥65 years | 0.37 | -0.26, 1.0 | 0.2 |
| Female sex | -0.50 | -1.1, 0.14 | 0.13 |
| (R-)ISS Stage III | -0.31 | -0.99, 0.38 | 0.4 |
| ≥VGPR after induction | -0.54 | -1.2, 0.13 | 0.11 |

**Table S5.11.** Time to platelet recovery, linear regression model.

| Parameter | Beta | 95% CI | *p*-Value |
|---|---|---|---|
| Daratumumab used | 2.0 | 1.0, 3.0 | <0.001 |
| Age ≥65 years | 0.33 | -0.59, 1.2 | 0.5 |
| Female sex | -0.95 | -1.9, -0.04 | 0.042 |
| (R-)ISS Stage III | 0.15 | -0.87, 1.2 | 0.8 |
| ≥VGPR after induction | 0.10 | -0.88, 1.1 | 0.8 |

**Table S5.12.** ≥1 platelet concentrate used (yes/no), logistic regression model.

| Parameter | OR | 95% CI | *p*-Value |
|---|---|---|---|
| Daratumumab used | 3.63 | 0.50, 75.2 | 0.3 |
| Age ≥65 years | 3.55 | 0.51, 71.8 | 0.3 |
| Female sex | 0.74 | 0.12, 4.45 | 0.7 |
| (R-)ISS Stage III | 0.69 | 0.12, 5.41 | 0.7 |
| ≥VGPR after induction | 0.00 | | >0.9 |

**Table S5.13.** Number of platelet concentrates transfused, linear regression model.

| Parameter | Beta | 95% CI | *p*-Value |
|---|---|---|---|
| Daratumumab used | 1.9 | 0.89, 3.0 | <0.001 |
| Age ≥65 years | 0.67 | -0.30, 1.6 | 0.2 |
| Female sex | -0.96 | -1.9, 0.02 | 0.055 |
| (R-)ISS Stage III | -0.05 | -1.1, 1.0 | >0.9 |
| ≥VGPR after induction | -0.32 | -1.4, 0.71 | 0.5 |

**Table S5.14.** ≥1 erythrocyte concentrate transfused (yes/no), logistic regression model.

| Parameter | OR | 95% CI | *p*-Value |
|---|---|---|---|
| Daratumumab used | 2.26 | 0.99, 5.43 | 0.058 |
| Age ≥65 years | 1.30 | 0.62, 2.76 | 0.5 |
| Female sex | 1.54 | 0.73, 3.32 | 0.3 |
| (R-)ISS Stage III | 1.74 | 0.75, 4.23 | 0.2 |
| ≥VGPR after induction | 0.79 | 0.35, 1.74 | 0.6 |

**Table S5.15.** Number of erythrocyte concentrates transfused, linear regression model.

| Parameter | Beta | 95% CI | *p*-Value |
|---|---|---|---|
| Daratumumab used | 0.55 | -0.35, 1.4 | 0.2 |
| Age ≥65 years | 0.60 | -0.20, 1.4 | 0.14 |
| Female sex | -0.37 | -1.2, 0.44 | 0.4 |
| (R-)ISS Stage III | -0.20 | -1.1, 0.73 | 0.7 |
| ≥VGPR after induction | -0.38 | -1.3, 0.52 | 0.4 |

**Table S5.16.** ≥1 infectious complication (yes/no), logistic regression model.

| Parameter | OR | 95% CI | *p*-Value |
|---|---|---|---|
| Daratumumab used | 1.94 | 0.86, 4.57 | 0.12 |
| Age ≥65 years | 1.93 | 0.92, 4.21 | 0.088 |
| Female sex | 1.17 | 0.55, 2.50 | 0.7 |
| (R-)ISS Stage III | 1.54 | 0.68, 3.65 | 0.3 |
| ≥VGPR after induction | 0.91 | 0.41, 2.00 | 0.8 |

**Table S6.** Basal and treatment response characteristics of patients treated with RVd vs D-RVd and mobilized with gemcitabine + G-CSF.

| Parameter | RVd (*n*=27) | D-RVd (*n*=22) | *p*-Value |
|---|---|---|---|
| Age at diagnosis (y), median (range) | 61 (46-72) | 57.5 (47-71) | 0.0806 |
| Male sex, *n* (%) | 17 (63) | 11 (50) | 0.3984 |
| FISH, *n* (%)[a] | | | >0.9999 |
| High risk cytogenetics | 7 (39) | 7 (37) | |
| Non-high risk | 11 (61) | 12 (63) | |
| (R-)ISS, *n* (%)[b] | | | 0.6134 |
| I | 9 (35) | 6 (27) | |
| II | 9 (35) | 11 (50) | |
| III | 8 (31) | 5 (23) | |
| Induction cycles before apheresis, *n* (%) | | | 0.1996 |
| 3 | 6 (22) | 2 (9) | |
| 4 | 18 (67) | 20 (91) | |
| 5 | 2 (7) | 0 (0) | |
| 6 | 1 (4) | 0 (0) | |
| HDCT, *n* (%) | | | 0.6854 |
| Treosulfan/Melphalan | 24 (89) | 18 (82) | |
| Melphalan | 3 (11) | 4 (18) | |
| ≥VGPR at Mobilisation, *n* (%)[c] | 19 (73) | 14 (70) | >0.9999 |
| ≥CR after ASCT, *n* (%)[d] | 19 (70) | 19 (90) | 0.1518 |
| MRD negativity, *n* (%)[e] | 16 (62) | 16 (80) | 0.2121 |

Data missing for *n* patients (RVd/D-RVd): [a](9/3), [b](1/0), [c](1/2), [d](0/1), [e](1/2).

**Table S7.** Mobilization and hematologic recovery metrics in patients treated with RVd vs D-RVd and mobilized with gemcitabine + G-CSF.

| Parameter | RVd (*n*=27) | D-RVd (*n*=22) | *p*-Value |
|---|---|---|---|
| Apheresis on the planned date, *n* (%) | 12 (44) | 6 (27) | 0.2481 |
| Mobilization days until apheresis (d), median (range) | 9 (8-10) | 9 (8-10) | 0.0568 |
| Plerixafor used, *n* (%)[a] | 5 (22) | 10 (50) | 0.0642 |
| Measurements at day of apheresis: | | | |
| CD34+ × $10^6$/L, median (range)[b] | 54 (13.81-122.14) | 35.23 (6.05-106) | 0.0714 |
| WBC × $10^9$/L, median (range)[c] | 34.5 (13.91-53.01) | 37.03 (18.41-75.49) | 0.3146 |
| CD34+/WBC (%), median (range)[c] | 0.16 (0.04-0.38) | 0.1 (0.02-0.27) | 0.0118 |
| Apheresis time (min), median (range)[d] | 272 (121-541) | 359.5 (192-1000) | 0.0331 |
| coll. CD34+ × $10^6$/kg BW, median (range) | 9.42 (2.6-17.13) | 8.16 (3.26-13.66) | 0.1164 |
| transp. CD34+ × $10^6$/kg BW, median (range) | 3.42 (2.05-5.37) | 3.16 (1.9-4.98) | 0.5300 |
| Hospitalization duration (d), median (range) | 23 (17-39) | 24.5 (19-39) | 0.6783 |
| Time to neutrophil recovery (d), median (range) | 11 (11-27) | 12 (10-20) | 0.0759 |
| Time to platelet recovery (d), median (range)[e] | 15 (12-20) | 16 (13-27) | 0.0237 |
| ≥1 PC used, *n* (%)[c] | 25 (93) | 22 (100) | 0.4949 |
| Number of PCs used, median (range) | 3 (1-16) | 5 (1-19) | 0.0223 |
| ≥1 EC used, *n* (%)[f] | 16 (59) | 19 (90) | 0.0222 |
| Number of ECs used, median (range)[g] | 2 (1-19) | 2 (1-8) | 0.9934 |
| Fever during hospitalization, *n* (%) | 27 (100) | 22 (100) | >0.9999 |
| ≥1 Infectious complication, *n* (%) | 13 (48) | 16 (73) | 0.1433 |

Data missing for *n* patients (RVd/D-RVd): [a](4/2), [b](2/0), [c](3/1), [d](1/4), [e](3/1), [f](0/1), [g](2/3).

**Table S8.** Basal and treatment response characteristics of patients treated with RVd vs D-RVd and mobilized with vinorelbine + G-CSF.

| Parameter | RVd (*n*=41) | D-RVd (*n*=21) | *p*-Value |
|---|---|---|---|
| Age at diagnosis (y), median (range) | 61 (36-75) | 60 (41-75) | 0.4618 |
| Male sex, *n* (%) | 29 (71) | 6 (29) | 0.0026 |
| FISH, *n* (%)[a] | | | 0.7561 |
| High risk cytogenetics | 9 (26) | 6 (32) | |
| Non-high risk | 25 (74) | 13 (68) | |
| (R-)ISS, *n* (%) | | | 0.2399 |
| I | 12 (29) | 7 (33) | |
| II | 22 (54) | 7 (33) | |
| III | 7 (17) | 7 (33) | |
| Induction cycles before apheresis, *n* (%) | | | 0.1111 |
| 3 | 11 (27) | 4 (19) | |
| 4 | 29 (71) | 16 (76) | |
| 5 | 1 (2) | 1 (5) | |
| 6 | 0 (0) | 0 (0) | |
| HDCT, *n* (%) | | | 0.2141 |
| Treosulfan/Melphalan | 38 (93) | 18 (81) | |
| Melphalan | 3 (7) | 4 (19) | |
| ≥VGPR at Mobilisation, *n* (%)[b] | 23 (61) | 17 (89) | 0.0322 |
| ≥CR after ASCT *n* (%) | 33 (80) | 20 (95) | 0.1498 |
| MRD negativity, *n* (%)[c] | 23 (61) | 19 (95) | 0.0053 |

Data missing for *n* patients (RVd/D-RVd): [a](7/2), [b](3/2), [c](3/1).

**Table S9.** Mobilization and hematologic recovery metrics in patients treated with RVd vs D-RVd and mobilized with vinorelbine + G-CSF.

| Parameter | RVd (*n*=41) | D-RVd (*n*=21) | *p*-Value |
|---|---|---|---|
| Apheresis on the planned date, *n* (%) | 34 (83) | 13 (62) | 0.1150 |
| Mobilization days until apheresis (d), median (range) | 8 (8-10) | 8 (8-9) | 0.1150 |
| Plerixafor used, *n* (%)[a] | 7 (23) | 4 (24) | >0.9999 |
| Measurements at day of apheresis: | | | |
| CD34+ × $10^6$/L, median (range)[b] | 73.69 (21-295.14) | 43.03 (19.97-115.6) | 0.0005 |
| WBC × $10^9$/L, median (range)[c] | 30.29 (8.97-63.72) | 26.82 (16.9-52.32) | 0.9518 |
| CD34+/WBC (%), median (range)[c] | 0.29 (0.07-0.83) | 0.16 (0.08-0.45) | 0.0005 |
| Apheresis time (min), median (range)[d] | 231 (99-660) | 277 (158-460) | 0.0371 |
| coll. CD34+ × $10^6$/kg BW, median (range) | 12.07 (3.35-41.54) | 8.27 (3.6-17.37) | 0.0007 |
| transp. CD34+ × $10^6$/kg BW, median (range) | 4.01 (2.43-10.36) | 3.3 (2-5.15) | 0.0029 |
| Hospitalization duration (d), median (range) | 22 (18-51) | 22 (18-35) | 0.6480 |
| Time to neutrophil recovery (d), median (range) | 11 (9-13) | 11(10-12) | 0.1339 |
| Time to platelet recovery (d), median (range)[e] | 13 (11-20) | 15 (11-25) | 0.0409 |
| ≥1 PC used, *n* (%)[f] | 38 (95) | 20 (95) | >0.9999 |
| Number of PCs used, median (range)[g] | 2 (1-8) | 2.5 (1-8) | 0.6538 |
| ≥1 EC used, *n* (%)[h] | 20 (53) | 11 (55) | >0.9999 |
| Number of ECs used, median (range)[i] | 1 (1-4) | 1 (1-4) | 0.3108 |
| Fever during hospitalization, *n* (%) | 37 (90) | 20 (95) | 0.6541 |
| ≥1 Infectious complication, *n* (%) | 24 (59) | 13 (62) | >0.9999 |

Data missing for *n* patients (RVd/D-RVd): [a](10/4), [b](3/1), [c](6/3), [d](5/2), [e](2/0), [f](1/0), [g](3/0), [h](3/1), [i](6/3).

**Table S10.** Comparison between basal and treatment response characteristics of patients treated with D-RVd patients and mobilized with gemcitabine + G-CSF vs vinorelbine + G-CSF.

| Parameter | Gemcitabine (*n*=22) | Vinorelbine (*n*=21) | *p*-Value |
|---|---|---|---|
| Age at diagnosis (y), median (range) | 57.5 (47-71) | 60 (41-75) | 0.8238 |
| Male sex, *n* (%) | 11 (50) | 6 (29) | 0.2152 |
| FISH, *n* (%)[a] | | | >0.9999 |
| High risk cytogenetics | 7 (37) | 6 (32) | |
| Non-high risk | 12 (63) | 13 (68) | |
| (R-)ISS, *n* (%) | | | 0.5459 |
| I | 6 (27) | 7 (33) | |
| II | 11 (50) | 7 (33) | |
| III | 5 (23) | 7 (33) | |
| Induction cycles before apheresis, *n* (%) | | | 0.1955 |
| 3 | 2 (9) | 4 (19) | |
| 4 | 20 (91) | 16 (76) | |
| 5 | 0 (0) | 1 (5) | |
| 6 | 0 (0) | 0 (0) | |
| Start of Daratumumab (Cycle), *n* (%) | | | >0.9999 |
| 1 | 12 (55) | 13 (62) | |
| 2 | 8 (36) | 7 (33) | |
| 3 | 1 (5) | 1 (5) | |
| 4 | 1 (5) | 0 (0) | |
| HDCT, *n* (%) | | | >0.9999 |
| Treosulfan/Melphalan | 18 (82) | 18 (81) | |
| Melphalan | 4 (18) | 4 (19) | |
| ≥VGPR at Mobilisation, *n* (%)[b] | 14 (70) | 17 (89) | 0.2351 |
| ≥CR after ASCT *n* (%)[c] | 19 (90) | 20 (95) | >0.9999 |
| MRD negativity, *n* (%)[d] | 16 (80) | 19 (95) | 0.3416 |

Data missing for *n* patients (Gemcitabine/Vinorelbine): [a](3/2), [b](2/2), [c](1/0), [d](2/1).

**Disclaimer/Publisher's Note:** The statements, opinions and data contained in all publications are solely those of the individual author(s) and contributor(s) and not of MDPI and/or the editor(s). MDPI and/or the editor(s) disclaim responsibility for any injury to people or property resulting from any ideas, methods, instructions or products referred to in the content.
